# TiO_2_-Photocatalyst-Induced Degradation of Dog and Cat Allergens under Wet and Dry Conditions Causes a Loss in Their Allergenicity

**DOI:** 10.3390/toxics11080718

**Published:** 2023-08-21

**Authors:** Ryosuke Matsuura, Arisa Kawamura, Rizo Ota, Takashi Fukushima, Kazuhiro Fujimoto, Masato Kozaki, Misaki Yamashiro, Junichi Somei, Yasunobu Matsumoto, Yoko Aida

**Affiliations:** 1 Laboratory of Global Infectious Diseases Control Science, Graduate School of Agricultural and Life Sciences, The University of Tokyo, 1-1-1 Yayoi, Bunkyo-ku, Tokyo 113-8657, Japan; matsuura-ryosuke@g.ecc.u-tokyo.ac.jp (R.M.);; 2 Inuyama Animal General Medical Center, 29 Oomishita, Haguro, Inuyama 484-0894, Japan; 3 Kaltech Corporation, Hirotake Bldg. 3-3-7 Bakuromachi, Chuo-ku, Osaka 541-0059, Japan; 4 Laboratory of Global Animal Resource Science, Graduate School of Agricultural and Life Sciences, The University of Tokyo, 1-1-1 Yayoi, Bunkyo-ku, Tokyo 113-8657, Japan

**Keywords:** Can f1, Fel d1, TiO_2_ photocatalyst, animal allergen, antigenicity, allergen degradation

## Abstract

Allergies to dogs and cats can cause enormous damage to human health and the economy. Dog and cat allergens are mainly found in dog and cat dander and are present in small particles in the air and in carpets in homes with dogs and cats. Cleaning houses and washing pets are the main methods for reducing allergens in homes; however, it is difficult to eliminate them completely. Therefore, we aimed to investigate whether a TiO_2_ photocatalyst could degrade dog and cat allergens. Under wet conditions, exposure to the TiO_2_ photocatalyst for 24 h degraded Can f1, which is a major dog allergen extracted from dog dander, by 98.3%, and Fel d1, which is a major cat allergen extracted from cat dander, by 93.6–94.4%. Furthermore, under dry conditions, the TiO_2_ photocatalyst degraded Can f1 and Fel d1 by 92.8% and 59.2–68.4%, respectively. The TiO_2_ photocatalyst abolished the binding of dog and cat allergens to human IgE by 104.6% and 108.6%, respectively. The results indicated that the TiO_2_ photocatalyst degraded dog and cat allergens, causing a loss in their allergenicity. Our results suggest that TiO_2_ photocatalysis can be useful for removing indoor pet allergens and improving the partnership between humans and pets.

## 1. Introduction

Allergic diseases such as rhinoconjunctivitis and asthma are associated with human health problems worldwide. Allergic diseases have been classified as asthma, rhinitis, conjunctivitis, dermatitis, urticaria, food hypersensitivity, drug hypersensitivity, insect sting or bite hypersensitivity, and anaphylaxis by the World Allergy Organization based on the mechanisms that initiate and mediate allergic reactions [1]. Allergic diseases cause significant economic losses [2]. For instance, they contribute to the highest financial burden in the USA, with an estimated loss of productivity of USD 593 per employee annually [3]. In addition, the loss of productivity due to rhinitis was estimated to be 2.7 billion EUR annually [4]. Notably, dogs and cats are the most prevalent household pets worldwide and, hence, are the most common source of pet allergens causing allergic diseases, burdening the health sector. Further, the population of pet dogs and cats has also been increasing in Europe [5,6]. Notably, as animal therapy is attracting attention in the field of human medicine, the importance of pet dogs and cats is expected to increase in the future [7]. However, it is estimated that 10–20% of the global population is allergic to dogs and cats, and this proportion is further increasing [2]. In the USA, the cost of caring for acute asthma in dog-allergic patients is estimated to add USD 500 million–1 billion [8]. In addition, allergies to cats are one of the reasons for rehoming pet cats [9]. Therefore, it is important to establish a method for suppressing pet allergens to enhance the bonding between pets and humans.

Seven (Can f1–Can f7) and eight (Fel d1–Fel d8) proteins have been identified as dog and cat allergens, respectively [2]. Dog and cat allergens have been detected in serum, dander (skin and fur), hair, saliva, and urine [2,10]. Additionally, cat allergens have also been detected in the anal and sebaceous glands [2,11]. These allergens are mainly detected on carpets and upholstery, which are a known reservoir of allergens in homes [11,12]. Notably, dog and cat allergens are suspended in the air for a long time [13,14]. Therefore, several methods for reducing allergens have been investigated. The most efficient methods include washing the animals and cleaning rooms. First, washing dogs and cats twice a week significantly reduces allergen levels at home [15]. Second, vacuuming and washing carpets decrease cat allergens [12]. Although these methods can significantly reduce the number of allergens, some allergens still remain. To improve cleaning efficiency, sodium hypochlorite was studied for the degradation of recombinant Fel d1 [16]. However, it is not suitable for reducing airborne allergens because of its toxicity. Finally, hypoallergenic dogs and cats have been investigated to reduce allergic diseases. One approach has been to vaccinate cats to induce neutralizing antibodies against Fel d1 and reduce its secretion [17]. However, there are ethical problems associated with vaccines that have no direct benefit for animals [2]. Dog breeds with little hair loss or long hair, such as Labradors and poodles, are thought to be hypoallergenic breeds that release fewer allergens into the environment [18]. However, floor and airborne allergen levels from these hypoallergenic dog breeds are similar to those of non-hypoallergenic dog breeds [18]. These studies suggest that a novel approach is required to effectively reduce pet allergens in the environment.

Photocatalysts can degrade organic matter while being harmless to human health. Many compounds such as titanium dioxide (TiO_2_), tungsten trioxide (WO_3_), iron (III) oxide (Fe_2_O_3_), and graphitic carbon nitride (CN) are known to exhibit photocatalysis and are being actively researched [19,20,21,22]. Photocatalysts are excited by light and exhibit strong oxidation–reduction reactions, generating reactive oxygen species (ROS) such as hydroxyl (^−^OH) and superoxide radicals (O_2_^−^) [23]. Hydrogen production via this photocatalytic oxidation–reduction reaction is attracting a great deal of attention in the production of clean energy [24]. Additionally, ROS produced by photocatalysts kill microorganisms, such as *Escherichia coli*, *Bacillus cereus*, *Enterobacter* spp., *Porphyromonas gingivalis*, *Salmonella* spp., *Vibrio parahaemolyticus*, and *Legionella pneumophila* [25,26,27,28,29,30,31,32,33,34,35,36,37], and inactivate viruses such as influenza, hepatitis C, vesicular stomatitis, enterovirus, herpes, Zika, human coronavirus, bovine coronavirus, human norovirus, murine norovirus, severe acute respiratory syndrome (SARS)-associated coronavirus, bacteriophage, and SARS-CoV2 [19,20,38,39,40,41,42,43,44,45]. In particular, our previous study showed that a TiO_2_ photocatalyst, which is the most common photocatalyst, inactivates SARS-CoV-2 in aerosols [19], suggesting that it is effective for inactivating organic matter suspended in the air. In addition, photocatalysts can degrade viral proteins and endotoxins, which are the lipopolysaccharides on the outer membrane of Gram-negative bacteria [19,36]. These results suggest that photocatalysts can degrade high-molecular-weight organic matter, such as animal allergens. Moreover, the TiO_2_ photocatalyst degrades the pollen antigens of *Asteraceae*, *Poaceae*, *Cupressus,* and *Platanus* [46,47,48]. Interestingly, ovalbumin treated with a TiO_2_ photocatalyst showed lower allergenicity in mice [49]. To increase the efficiency of this photocatalytic reaction, co-catalysts for accurately controlling charge separation were actively explored [24,50]. Indeed, integration of an MXene in CN can trap the electrons to reduce the energy loss and enlarge the light absorption range of CN, which greatly improves the energy utilization efficiency [22]. Platinum (Pt) is also a known co-catalyst for TiO_2_ and the formation of a Ti^3+^ state on the TiO_2_ surface close to the Pt nanoparticles and a Pt^δ+^ state on the Pt nanoparticles [51]. Additionally, TiO_2_ photocatalysts do not harm human health. Although the International Agency for Research on Cancer classified TiO_2_ as possibly carcinogenic to humans (group 2B), it noted that there was limited evidence of carcinogenicity in experimental animals. Additionally, no increased risks of total cancers, including lung cancer and other causes of death, were found in workers employed in the TiO_2_ production industry in the USA and Europe [52,53]. These results strongly suggest that there is very little risk from the inhalation of suspended TiO_2_ particles. Furthermore, the band gap energy and peak photoexcitation wavelength of rutile-type TiO_2_ have been determined to be 3.05 eV and 406.5 nm, respectively [54]. Rutile-type TiO_2_ can be excited by a 405 nm light-emitting diode (LED), which is an inexpensive and harmless light source compared to ultraviolet C [19]. Therefore, the use of TiO_2_ photocatalysts is an attractive choice for reducing the number of allergens in the air, keeping indoor environments cleaner. However, the use of TiO_2_ photocatalysts to degrade dog and cat allergens has not yet been demonstrated.

In this study, we aimed to investigate whether rutile-type TiO_2_/PtO_2_ (hereafter referred to as “TiO_2_”)-coated glass sheets exerted photocatalytic activity following excitation by light with a wavelength of 405 nm. Furthermore, we aimed to investigate whether TiO_2_ photocatalysts could degrade dog and cat allergens in wet and dry conditions, respectively. We hypothesized that TiO_2_ photocatalysts could degrade dog and cat allergens and decrease their allergenicity.

## 2. Materials and Methods

### 2.1. Preparation of TiO_2_-Coated Glass Sheet

TiO_2_ was coated onto a glass sheet (XU1310010; Osaka Lighting Corp., Osaka, Japan) as follows: A commercial powder of fine (approximately 20 nm) rutile-type TiO_2_ containing approximately 1% platinum dioxide was dispersed into ion-exchanged water (to improve the photocatalytic reaction) (MPT-623, Ishihara Sangyou Kaisha, Ltd., Osaka, Japan) [55,56], followed by the immersion of a glass sheet in the water. The glass sheet was then air dried at room temperature prior to being calcined in air for 90 min at 400 °C, which did not change the crystal structure of the rutile-type TiO_2_ [57].

### 2.2. Transmission Electron Microscope (TEM)

To observe the TiO_2_ particle, a TEM image was obtained using a Titan™ 80-300 electron microscope (FEI-Company, Hillsboro, OR, USA).

### 2.3. TiO_2_ Photocatalyst Treatment

**Figure 1:**
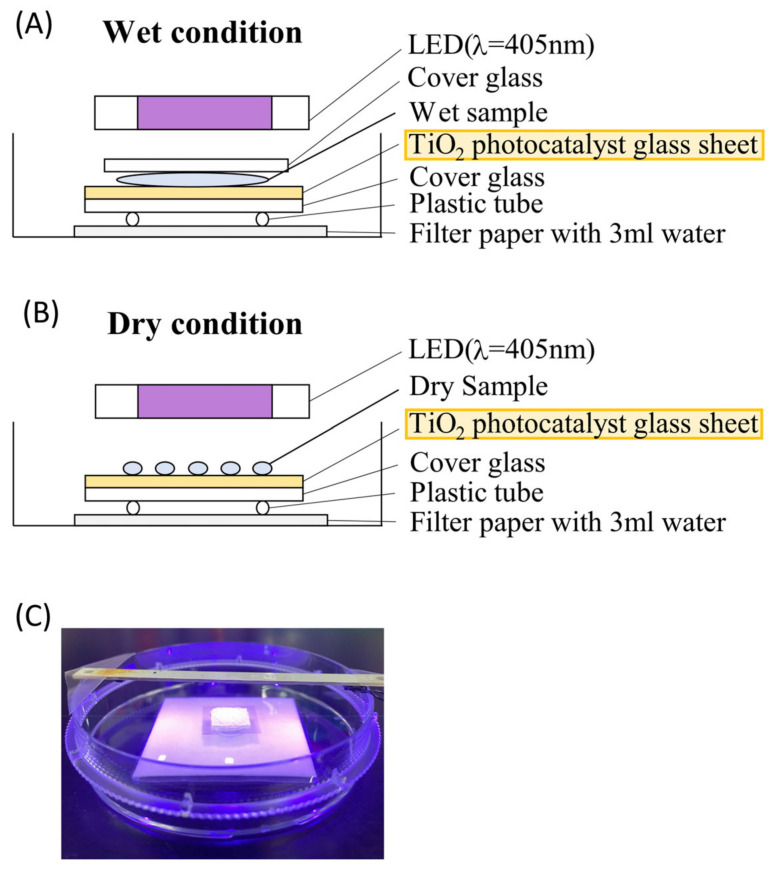
Schematic diagram (**A**,**B**) and images (**C**) of the TiO_2_ photocatalytic reaction system. (**A**,**B**): Wet filter paper was placed in a 10 cm dish to avoid dryness. A TiO_2_-coated glass sheet (1 cm × 1 cm) and a non-coated glass sheet were placed on a cover glass, which was on the plastic tube that was later placed on the filter paper. (**A**): To confirm the photocatalytic effect in wet conditions, 100 μL of wet sample solution was placed on the TiO_2_-coated glass sheet and the non-coated glass sheet and the wet sample was covered with the cover glass. (**B**): To confirm the photocatalytic effect in dry conditions, 6 mg of freeze-dried allergen was placed on the TiO_2_-coated glass sheet and the non-coated glass sheet. (**C**): The TiO_2_ photocatalyst was excited by LED light (wavelength 405 nm) for up to 24 h. After the photocatalytic reaction, samples were collected by adding either 100 μL PBS or 100 μL water in wet and in dry conditions, respectively.

The photocatalytic reaction was performed according to JIS R1752:2020 [58], with minor modifications (Figure 1). Briefly, under wet conditions, filter paper was placed at the bottom of a 10 cm dish and damped with 3 mL of distilled water to preserve moisture (Figure 1A). To avoid direct contact with the filter paper, a cover glass and plastic tube were placed on the filter paper, and a glass sheet (1 cm × 1 cm) coated with TiO_2_ was placed on top of the cover glass (Figure 1A). In the “TiO_2_ + Light” group, 100 μL of the sample solution was placed on the TiO_2_-coated glass sheet, covered by a cover glass (Figure 1A), and the TiO_2_ photocatalyst was excited by 405 nm LED source irradiation (Figure 1C). Under dry conditions, in the “TiO_2_ + Light” group, 6 mg of the dry sample was placed on a TiO_2_-coated glass sheet (Figure 1B), and the TiO_2_ photocatalyst was excited using LED light (405 nm) source irradiation (Figure 1C). The controls included a dry sample incubated with either a TiO_2_-coated glass sheet without LED light (“TiO_2_ in dark” group) or both a glass and LED light (“Glass + Light” group), or a glass without LED light (“Glass in dark” group).

### 2.4. Methylene Blue Degradation

To investigate the effect of the TiO_2_ photocatalyst on methylene blue, 100 μL of 12.5 μM methylene blue solution was applied once and adsorbed onto the TiO_2_-coated glass sheet and then irradiated using 405 nm LED light for 0, 10, 20, 30, 40, 50, and 60 min, as shown in Figure 1A,C. The resulting methylene blue solution was collected at each time point by washing and immersing it in 100 μL of distilled water. Next, the absorbance at 660 nm was measured using an Ensight Perkin Elmer multimode plate reader (Perkin Elmer, Milan, Italy). The rate of methylene blue degradation was calculated from the exponential regression of OD_660_ versus the photocatalytic reaction time.

### 2.5. Analysis of the Degradation of Animal Allergens by TiO_2_ Photocatalyst under Wet and Dry Conditions Using Western Blot

To confirm the degradation of animal allergens by the TiO_2_ photocatalyst under wet conditions, 12 mg of freeze-dried crude antigen extracted from dog and cat hair coat and epithelium (Institute of Tokyo Environmental Allergy, Tokyo, Japan) was diluted in 1 mL of distilled water, and 100 μL of the sample was treated with the TiO_2_ photocatalyst for up to 24 h. After the photocatalytic reaction, the sample was collected following the addition of 100 μL of phosphate-buffered saline (PBS) at each time point.

To confirm the degradation of animal allergens by the TiO_2_ photocatalyst under dry conditions, 6 mg of freeze-dried crude antigen extracted from dog and cat hair coat and epithelium was treated with the TiO_2_ photocatalyst for up to 24 h. At each time point, the sample was collected by adding 100 μL distilled water.

To confirm whether the TiO_2_ photocatalyst degraded animal allergens, 15 µL of the above-mentioned collected samples were mixed with 5 µL of sample buffer (0.15 M Tris–HCl, 10% sodium dodecyl sulfate (SDS), 30% glycerol, and 0.5% bromophenol blue) and heated at 100 °C for 5 min. A cat allergen sample under dry conditions was additionally diluted twice to avoid saturation. Thereafter, the 20 µL sample was loaded on a 15% SDS–polyacrylamide gel for electrophoresis using a running buffer containing 0.3% Tris, 0.1% SDS, and 1.44% glycine. Proteins were then transferred onto polyvinylidene difluoride membranes (Millipore, Billerica, MA, USA) using a Trans-Blot Turbo apparatus (Bio-Rad, Hercules, CA, USA). The membranes were blocked with 5% non-fat skim milk and then incubated overnight with anti-Can f1 monoclonal antibodies (Mab; 10D4) (1:500; Indoor Biotechnologies, Charlottesville, VA, USA) and anti-Fel d1 Mab (6F9) (1:100; Indoor Biotechnologies) at 4 °C. After washing with PBS containing 1% TWEEN 20 (Nacalai Tesque Inc., Kyoto, Japan), the membranes were incubated with horseradish-peroxidase-conjugated goat anti-mouse immunoglobulin (Ig) G (1:2000; Jackson ImmunoResearch, West Grove, PA, USA) at room temperature for 30 min. Following treatment with SuperSignal West Pico PLUS Chemiluminescent Substrate (Thermo Fisher Scientific, Waltham, MA, USA), the signals were visualized. Images were acquired using the WSE-6100 LuminoGraph I (ATTO CORPORATION, Tokyo, Japan). The intensity of the bands was analyzed using ImageJ software (National Institutes of Health, Bethesda, MD, USA).

### 2.6. Allergenicity Detection of Animal Allergens by Enzyme-Linked Immunosorbent Assay (ELISA)

To confirm whether the TiO_2_ photocatalyst decreased the allergenicity of animal allergens, 100 μL of biotinylated dog and cat dander allergens (Nippon Chemiphar, Tokyo, Japan) were treated with the TiO_2_ photocatalyst for up to 24 h. Samples were collected by adding 100 μL of PBS at each time point. Allergenicity was then detected by ELISA using a specific IgE control containing human IgE anti-dog and cat allergen by Oriton IgE CHEMIPHAR (Nippon Chemiphar, Tokyo, Japan) according to the manufacturer’s instructions. The intensity of the membrane was analyzed using ImageJ software.

### 2.7. Statistical Analysis

Statistical comparisons were performed using Student’s *t*-test and two-way analysis of variance with Dunnett’s test. Statistical significance was set at *p* < 0.05. All calculations were performed using R software (version 3.6.3; R Foundation for Statistical Computing, Vienna, Austria).

## 3. Results

### 3.1. Properties of TiO_2_ and Degradation of Methylene Blue by TiO_2_

**Figure 2:**
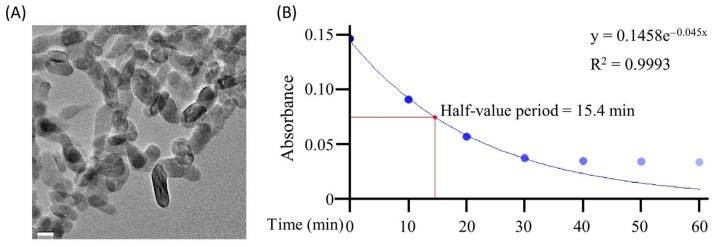
TEM image of the TiO_2_ particles and methylene blue degradation by the TiO_2_ photocatalyst. (**A**): The TEM images show the TiO_2_ particles. Bar = 20 nm. (**B**): To confirm the ability of the TiO_2_ photocatalyst to decompose organic matter, 100 μL of 12.5 μM of methylene bule was treated with the TiO_2_ photocatalyst. Methylene blue was collected every 10 min, and the absorbance at 660 nm was measured. The degradation speed of methylene blue was calculated using exponential regression analysis, which evaluated methylene blue absorbance and irradiation time from 0 to 60 min before flattening out. R^2^ indicates the coefficient of determination.

TEM was used to analyze the TiO_2_ particle. A particle (approx. 20 nm in size) was observed (Figure 2A). These small particles were assumed to provide sufficient surface area and were expected to have high photocatalytic activity, which was evaluated by the degradation rate of methylene blue. As shown in Figure 2B, methylene blue was degraded in a time-dependent manner. The half-life was 15.4 min (R^2^ = 0.9993), and the curve flattened at an absorbance close to that of distilled water between 30 and 60 min after the photocatalytic reaction. These results suggest that TiO_2_ photocatalysts have a strong ability to decompose organic matter. The reaction rates were estimated using the following equation:

(1) $$r\;\left(\mu M/\min\right)=0.0107\;{\left[methylene\;blue\right]}^{0.992}$$

where *r* is the reaction rate, 0.0107 is the reaction rate constant (s^−1^), [*methylene blue*] is the concentration of methylene blue, and 0.992 is the order of the reaction. This reaction equation suggests that the degradation reaction was a first-order reaction. Therefore, the degradation of methylene blue depended only on its concentration, suggesting that the amount of titanium dioxide and the illuminance of the excitation light were sufficient for the decomposition of organic matter.

### 3.2. Dog Allergen Degradation by TiO_2_ Photocatalyst under Wet Conditions

**Figure 3:**
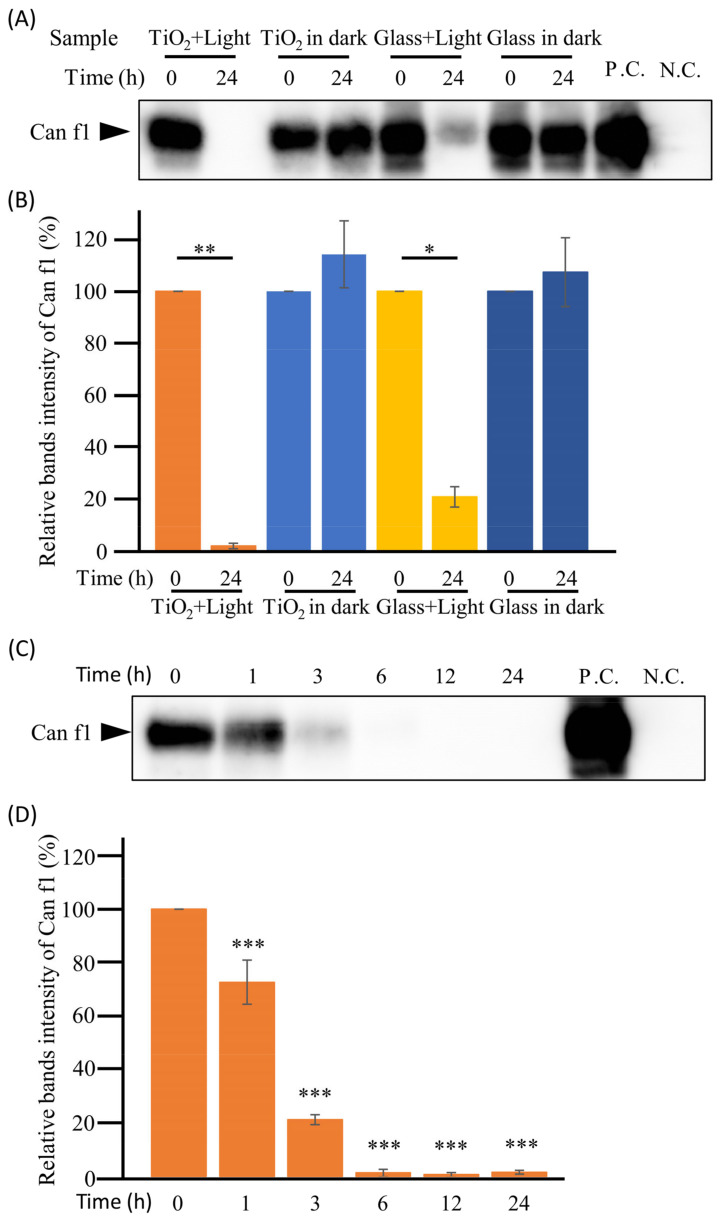
Degradation of dog allergens in wet conditions. (**A**): To confirm whether the TiO_2_ photocatalyst degraded dog antigens in wet conditions, 100 μL of crude antigen was extracted from the dog hair coat, and the epithelium (contained 10 μg/mL of Can f1) was treated with TiO_2_ photocatalyst for 0 or 24 h in the “TiO_2_ + Light” group. As a control, the crude dog antigen was incubated with either the TiO_2_-coated glass sheet without LED light (“TiO_2_ in dark” group) or both the glass and LED light (“Glass + Light” group), or the glass without LED light (“Glass in the dark” group). Further, 150 ng of crude Can f1 antigen and PBS were used as positive and negative controls (P.C. and N.C.), respectively. Can f1 was detected by Western blot using anti-Can f1 monoclonal antibody (10D4). The positions of Can f1 are indicated. Full-length blots are shown in Supplementary Figure S1. (**B**): The intensities of bands were analyzed using ImageJ software, and the quantitative results are shown in the bar diagram. (**C**): To confirm the time dependence of the photocatalytic degradation of dog allergens in wet conditions, 100 μL of crude dog antigen was treated with the TiO_2_ photocatalyst from 0 to 24 h, and Can f1 was detected by Western blot using anti-Can f1 monoclonal antibody (10D4). The positions of Can f1 are indicated. Full-length blots are shown in Supplementary Figure S2. (**D**): Intensities of bands were analyzed using ImageJ software, and the quantitative results are shown in the bar diagram. Each column and error bar represents the mean ± standard error (SD) of the two replicates. Significance between 0 h and other time points was determined using Student’s *t*-test or one-way analysis of variance followed by Dunnett’s multiple comparisons test. The asterisk indicates a significant difference (* *p* < 0.05; ** *p* < 0.01; *** *p* < 0.001).

As shown in Figure 3A, a 17 kDa band was identified as the Can f1 protein, which is the major dog allergen, by Western blot. Notably, this band completely disappeared in the “TiO_2_ + Light” group after 24 h of the TiO_2_ photocatalytic reaction. Moreover, the 24 h photocatalytic reaction significantly (*p* < 0.01) reduced the band intensity of Can f1 by 98.3% (Figure 3B). In the “Glass + Light” group, a faint band of Can f1 was observed; however, its band intensity was only 20.7%. In contrast, no decrease in band signal was detected either in the “TiO_2_ in dark” or “Glass in dark” groups. These results suggest that dog allergens were degraded by the TiO_2_ photocatalyst. However, the reduction in allergens due to light alone is thought to have been due to the denaturation of proteins by light and the accompanying heat.

To further confirm whether the photocatalytic reaction actually decomposed Can f1, crude dog antigen was treated with the TiO_2_ photocatalyst for 0 to 24 h. Figure 3C,D shows that the Can f1 band significantly (*p* < 0.001) decreased after 1 h in a time-dependent manner and completely disappeared after 6 h of the TiO_2_ photocatalytic reaction. These results suggest that the TiO_2_-coated glass sheets could degrade dog allergens under wet conditions.

### 3.3. Loss of Allergenicity of Dog Allergen by TiO_2_ Photocatalysis under Wet Conditions 

**Figure 4:**
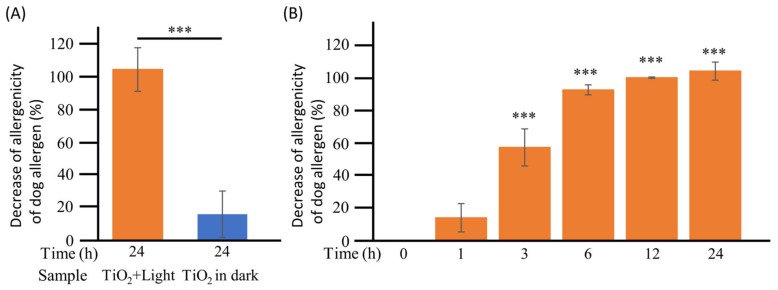
Detection of the allergenicity of the dog allergen in wet conditions. (**A**): To confirm the allergenicity of the dog allergen in wet conditions, 100 μL of biotinylated dog dander allergen was treated with TiO_2_ photocatalyst for 0 and 24 h, and its reactivity with human IgE was detected using Capture ELISA in the “TiO_2_ + Light group”. As a control, dog dander allergens were incubated with the TiO_2_-coated glass sheet without LED light (“TiO_2_ in dark” group). (**B**): To confirm the time dependence of the photocatalytic degradation of animal allergens in wet conditions, 100 μL of biotinylated dog dander allergen was treated with the TiO_2_ photocatalyst from 0 to 24 h. The dog allergen degradation ratio was calculated using the following equation: Decrease in the allergenicity of dog allergens (%) = (1 − intensity value at each time point/intensity value at 0 h) × 100. Each column and error bar represents the mean ± standard error (SD) of two or three replicates. Significance between 0 h and other time points was determined using Student’s *t*-test or one-way analysis of variance followed by Dunnett’s multiple comparisons test. The asterisk indicates a significant difference (*** *p* < 0.001).

As described above, the photocatalytic degradation of Can f1 was confirmed under wet conditions. However, it remained unclear whether this degradation caused a loss in its allergenicity. Therefore, the reactivity of biotinylated dog allergens to human IgE, which is involved in allergic reactions, was investigated using Capture ELISA (Figure 4). First, 100 μL of biotinylated dog allergen was treated with the TiO_2_ photocatalyst with or without excitation light for 0 and 24 h, and allergenicity was detected using human IgE. As shown in Figure 4A, the 24 h photocatalytic reaction completely (104.6%) abrogated the allergenicity of the dog dander allergen to human IgE. In contrast, only a 23.1% decrease in the allergenicity of the dog dander allergen to human IgE was observed after 24 h of incubation in the “TiO_2_ in dark” group. The level of allergenicity between the two groups that were treated with the TiO_2_ photocatalyst with or without LED showed significant differences (*p* < 0.001) (Figure 4A). Furthermore, as depicted in Figure 4B, the allergenicity of the dog dander allergen to human IgE significantly (*p* < 0.001) decreased after 3 h in a time-dependent manner and was undetectable after 12 h of TiO_2_ photocatalytic treatment. These results suggest that TiO_2_ photocatalysts induce the degradation of dog allergens, thereby reducing their allergenicity.

### 3.4. Cat Allergen Degradation by TiO_2_ Photocatalyst under Wet Conditions 

**Figure 5:**
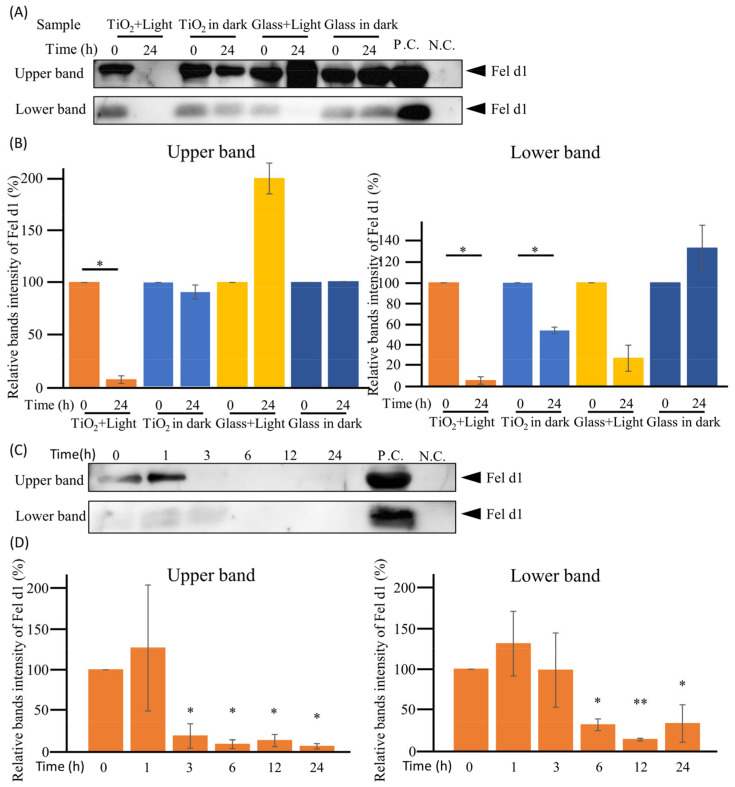
Degradation of cat allergens in wet conditions. (**A**): To confirm whether the TiO_2_ photocatalyst degrades cat antigens in wet conditions, 100 μL of crude antigen extracted from the cat hair coat and the epithelium (contained 10 μg/mL of Fel d1) was treated with the TiO_2_ photocatalyst for 0 or 24 h in the “TiO_2_ + Light group”. As a control, crude cat antigens were incubated with either a TiO_2_-coated glass sheet without LED light (“TiO_2_ in dark” group), both a glass and LED light (“Glass + Light” group), or a glass without LED light (“Glass in dark” group). Further, 150 ng of crude Fel d1 antigen and PBS were used as positive and negative controls (P.C. and N.C.), respectively. Fel d1 was detected by Western blot using anti-Fel d1 monoclonal antibody (6F9). Positions of the two Fel d1 bands are indicated. Full-length blots are shown in Supplementary Figure S3. (**B**): Intensities of the bands were analyzed using ImageJ software, and the quantitative results are shown in the bar diagram. (**C**): To confirm the time dependence of the photocatalytic degradation of the cat allergens in wet conditions, 100 μL of crude cat antigen was treated with TiO_2_ photocatalyst from 0 to 24 h. Fel d1 was detected by Western blot using anti-Fel d1 monoclonal antibody (6F9). Full-length blots are shown in Supplementary Figure S4. (**D**): Intensities of bands were analyzed using ImageJ software, and the quantitative results are shown in the bar diagram. Each column and error bar represents the mean ± standard error (SD) of two or three replicates. The significance between 0 h and other time points was determined using Student’s *t*-test or one-way analysis of variance followed by Dunnett’s multiple comparisons test. The asterisk indicates significant differences (* *p* < 0.05; ** *p* < 0.01).

Cat allergies are a major concern for human health. Therefore, the TiO_2_ photocatalytic effect on cat allergens under wet conditions was investigated. As shown in Figure 5A, two major bands of Fel d1 around 70 kDa (upper band) and 8 kDa (lower band), which were speculated to be a tetramer of Fel d1 and the chain 1 of Fel d1, respectively [11], were detected on the Western blots. Additionally, both bands completely disappeared after 24 h of the TiO_2_ photocatalytic treatment in the “TiO_2_ + Light” group. Notably, the 24 h photocatalytic reaction significantly (*p* < 0.05) reduced the band intensities of Fel d1 upper and lower bands by 93.6 and 94.4%, respectively (Figure 5B). In the “Glass + Light” group, the intensity of the lower band of Fel d1 decreased, whereas that of upper band increased, although non-significantly. This finding was attributed to protein denaturation and heat associated with light, which caused band smearing. In the “TiO_2_ in dark” group, the intensity of the lower band of Fel d1 also significantly (*p* < 0.05) decreased, indicating protein adsorption onto the photocatalyst. In contrast, no decrease was detected in the “Glass in dark” group. These results suggest that the cat allergen was degraded by the TiO_2_ photocatalyst.

To further confirm whether the photocatalytic reaction actually decomposed Fel d1, the crude cat antigen was treated with the TiO_2_ photocatalyst for 0–24 h. As shown in Figure 5C,D, the upper and lower bands of Fel d1 decreased significantly (*p* < 0.05 and *p* < 0.01) after 1 h in a time-dependent manner. These results suggest that the TiO_2_ photocatalysts could degrade both dog and cat allergens under wet conditions.

### 3.5. Loss of Allergenicity of Cat Allergen by TiO_2_ Photocatalysis under Wet Conditions 

**Figure 6:**
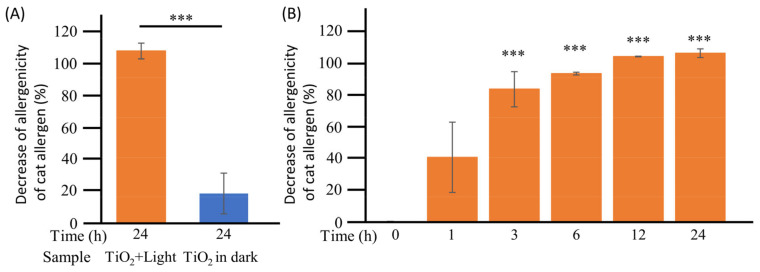
Detection of the allergenicity of the cat allergens in wet conditions. (**A**): To confirm the allergenicity of the cat allergens in wet conditions, 100 μL of biotinylated cat dander allergen was treated with the TiO_2_ photocatalyst for 0 and 24 h, and its reactivity with human IgE was detected using Capture ELISA in the “TiO_2_ + Light group”. As a control, cat dander allergen was incubated with a TiO_2_-coated glass sheet without LED light (“TiO_2_ in dark” group). (**B**): To confirm the time dependence of photocatalytic degradation of the cat allergens in wet conditions, 100 μL of biotinylated cat dander allergen was treated with the TiO_2_ photocatalyst from 0 to 24 h. The cat allergen degradation ratio was calculated using the following equation: Decrease in the allergenicity of cat allergens (%) = (1 − intensity value at each time point/intensity value at 0 h) × 100. Each column and error bar represents the mean ± standard error (SD) of two or three replicates. Significance between 0 h and other time points was determined using Student’s *t*-test or one-way analysis of variance followed by Dunnett’s multiple comparisons test. The asterisk indicates significant differences (*** *p* < 0.001).

As described above, the photocatalytic degradation of Fel d1 and Can f1 was confirmed under wet conditions. Next, to confirm the effect of the TiO_2_ photocatalyst on the allergenicity of cat allergens, the reactivity of the biotinylated cat allergen to human IgE was investigated. First, 100 μL of biotinylated cat allergen was treated with the TiO_2_ photocatalyst with or without irradiating excitation light for 0 and 24 h, and allergenicity was detected using human IgE. As shown in Figure 6A, the 24 h photocatalytic reaction completely (108.6%) reduced the allergenicity of cat dander allergens to human IgE. In contrast, only 18.7% of the allergenicity of cat dander allergen to human IgE was reduced after 24 h of incubation in the “TiO_2_ in dark” group. The level of allergenicity between the two groups that were treated by the TiO_2_ photocatalyst with or without LED light showed significant differences (*p* < 0.001) (Figure 6A). In addition, the allergenicity of the cat dander allergen to human IgE was significantly decreased by the TiO_2_ photocatalytic reaction after 3 h in a time-dependent manner and was undetectable after the 12 h TiO_2_ photocatalytic reaction (Figure 6B). These results suggest that cat allergens were also degraded by the TiO_2_ photocatalyst, thereby losing their allergenicity.

### 3.6. Cat Allergen Degradation by TiO_2_ Photocatalyst under Dry Conditions 

**Figure 7:**
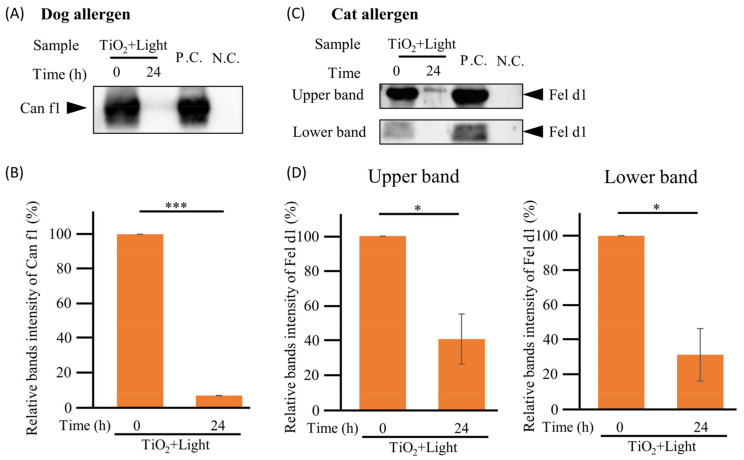
Degradation of dog and cat allergens in dry conditions. (**A**): To confirm whether the TiO_2_ photocatalyst degrades dog antigens in dry conditions, 6 mg of crude antigen extracted from the dog hair coat and the epithelium (contained 5 μg of Can f1) were treated with TiO_2_ photocatalyst for 0 or 24 h in the “TiO_2_ + Light” group. Further, 150 ng of crude Can f1 antigen and distilled water were used as positive and negative controls (P.C. and N.C.), respectively. The positions of Can f1 are indicated. Can f1 was detected by Western blot using anti-Can f1 monoclonal antibody (10D4). Full-length blots are shown in Supplementary Figure S5. (**B**): Intensities of bands were analyzed using ImageJ software, and the quantitative results are shown in the diagram. (**C**): To confirm whether the TiO_2_ photocatalyst degrades cat antigens in dry conditions, 6 mg of crude antigen extracted from the cat hair coat and the epithelium (contained 5 μg of Fel d1) were treated with the TiO_2_ photocatalyst for 0 or 24 h in the “TiO_2_ + Light” group. Further, 150 ng of crude Fel d1 antigen and distilled water were used as positive and negative controls (P.C. and N.C.), respectively. Fel d1 was detected by Western blot using anti-Fel d1 monoclonal antibody (6F9). The position of the two Fel d1 bands are indicated. Full-length blots are shown in Supplementary Figure S6. (**D**): Intensities of the bands were analyzed using ImageJ software, and the quantitative results are shown in the diagram. Each column and error bar represents the mean ± standard error (SD) of the three replicates. Significance between 0 and 24 h was determined using Student’s *t*-test. The asterisk indicates significant differences (* *p* < 0.05; *** *p* < 0.001).

In the real environment, animal allergens are present in upholstery such as carpets and in the air in a dry state, and cause allergies when inhaled. Therefore, we investigated the TiO_2_ photocatalytic degradation of animal allergens using 6 mg of freeze-dried crude dog antigens under dry conditions (Figure 1B) via Can f1 detection by Western blot. As shown in Figure 7A, the Can f1 band completely disappeared after 24 h of the TiO_2_ photocatalytic reaction under both dry and wet conditions. Notably, the 24 h photocatalytic reaction significantly (*p* < 0.001) reduced the band intensity of Can f1 by 92.8% (Figure 7B). These results suggest that TiO_2_ photocatalysts could degrade dog allergens via direct contact under dry conditions.

Similarly, we confirmed the TiO_2_ photocatalyst degradation of cat allergens under dry and wet conditions. The 6 mg of freeze-dried crude cat antigens was subjected to TiO_2_ photocatalytic reactions for 0 and 24 h (Figure 1B). As shown in Figure 7C, the intensity of the upper bands also decreased after 24 h of the TiO_2_ photocatalytic reaction under both conditions. Remarkably, the lower band of Fel d1 completely disappeared after 24 h of the TiO_2_ photocatalytic reaction under dry conditions. Notably, the 24 h photocatalytic reaction significantly (*p* < 0.05, upper band; *p* < 0.05, lower band) reduced the band intensity of Fel d1 by 59.2% and 68.4% in the upper and lower bands, respectively (Figure 7B). These results suggest that TiO_2_ photocatalysts could degrade cat allergens via direct contact under dry conditions.

## 4. Discussion

Here, we showed the TiO_2_ photocatalytic effect on dog and cat allergens. Notably, this is the first study to report that a TiO_2_ photocatalytic reaction for 24 h degraded 98.3% Can f1 and 93.6%–94.4% Fel d1 under wet conditions. Similar to this study, previous studies have confirmed a reduction in ovalbumin levels using Western blot [49]. In addition, morphological changes in pollen grains and chemical changes in the carbon and nitrogen bonds in allergenic pollen extracts by photocatalytic reactions have been reported [46,47,48]. These findings from previous studies strongly support our findings that dog and cat allergens are degraded by TiO_2_ photocatalysis. Furthermore, we found for the first time that 24 h of the TiO_2_ photocatalytic reaction degraded 92.8% Can f1 and 59.2–68.4% Fel d1 under dry conditions. In previous studies, only pollen grains were used for the study of photocatalytic degradation of allergens under dry conditions, and morphological changes in pollen grains and changes in the chemical bonds of carbon and nitrogen by photocatalytic reactions were confirmed [47,48]. This is the first study to confirm the direct degradation of allergen proteins under dry conditions. Our results provided evidence that the allergenicity of dog and cat allergens to human IgE decreased by 104.6% and 108.6%, respectively, after 24 h of the TiO_2_ photocatalytic reaction. Previous studies have shown that photocatalysts reduce the allergenicity of ovalbumin, which supports the results of the present study. This is the first study to clarify the effect of photocatalysts on the binding ability of human IgE to allergens. Conclusively, our findings suggest that TiO_2_ photocatalysis can reduce dog and cat allergens and promote a more hygienic space.

Our results clearly showed the degradation of Can f1, a major dog allergen, and Fel d1, a major cat allergen, by the TiO_2_ photocatalyst. Among human adults sensitized to dog allergens, up to 64% are Can f1 IgE-positive [59]. Similarly, up to 96% are Fel d1 IgE-positive among human adults sensitized to cat allergens [60]. Therefore, the effect of photocatalysts on the decomposition of these proteins is important for reducing dog and cat allergens. Additionally, TiO_2_ photocatalysis abolished the binding of dog and cat allergens in dander to human IgE. Dog dander contains not only Can f1 but also Can f2, Can f3, Can f4, Can f5, and Can f6 [61,62]. Cat dander also contains Fel d1 and Fel d4 [63]. Therefore, TiO_2_ photocatalysts might degrade not only Can f1 and Fel d1, but also other dog and cat allergens. Further studies are required to investigate whether TiO_2_ photocatalysts degrade other dog and cat allergens using specific antibodies.

The cat Fel d1 allergen was detected as two major bands at approximately 70 kDa (upper band) and 8 kDa (lower band). Previous reports have shown that various bands were detected in samples collected from different parts of the same individual using the anti-Fel d1 monoclonal antibody (10D4) used in this study. Therefore, multiple bands are characteristic of Fel d1 [64]. Notably, Fel d1 is a glycoprotein consisting of two identical heterodimers, each 18–19 kDa, which eventually form a tetramer [11,65,66]. Each dimer consists of two polypeptide chains, chain 1 and chain 2, having molecular weights of 8 kDa and 10 kDa, respectively [11,67,68]. Therefore, it was considered that the tetramer and chain 1 were detected in this study, and both were successfully degraded by the photocatalytic reaction. This suggests that the TiO_2_ photocatalyst is capable of degrading Fel d1 at various molecular weights.

The allergen reduction rate under dry conditions was lower than that under wet conditions. previous studies that have reported the decomposition of plastics due to the ROS generated by photocatalysts suggest that the hydration of photocatalysts to generate ROS is important for the decomposition of organic substances by photocatalysts [69,70]. Notably, the photocatalytic degradation of high-density polyethylene is more efficient in the aqueous state than in the solid state [69]. This may explain why photocatalysis under dry conditions was less efficient than under wet conditions in this study. Allergens in carpets are considered dry [12]. Airborne allergens adhere to small particles and float in the air [12,14]. Therefore, the fact that TiO_2_ photocatalysts can degrade dog and cat allergens under not only wet but also dry conditions strongly indicates the potential of TiO_2_ photocatalysts for the degradation of allergens, not only in experimental environments but also in actual environments.

It is well known that photocatalysts decompose organic matter and inactivate microorganisms and viruses [19,20,25,26,27,28,29,30,31,32,33,34,35,36,37,38,39,40,41,42,43,44,45]. Photocatalysts can also inactivate viruses in the air [19]. However, to the best of our knowledge, studies on whether photocatalysts can reduce allergens are limited [46,47,48,49]. In this study, we demonstrated for the first time the photocatalytic degradation of dog and cat allergens, which may benefit human health and the economy and reduce the rehoming of pets. However, one of the limitations of this study is that it was conducted in an experimental environment. Allergic rhinitis, bronchitis asthma, and conjunctivitis are particularly common symptoms of animal allergy. Since these are caused by inhalation through the mouth and nose, and via contact with the eyes, airborne allergens are thought to be one of the causes of animal allergy. Therefore, future studies to determine whether allergens in the actual environment can be removed using an air purifier equipped with a TiO_2_-coated glass sheet are urgently needed. Additionally, although photocatalysts are considered safe, a deeper simultaneous evaluation of their safety among people and pets, especially the inhalation of nanoparticles, will allow photocatalysis to contribute to the creation of more clean, safe, and secure spaces. Finally, we believe that this approach is the first step towards using photocatalysts as a tool to efficiently remove allergens and build a better partnership between humans and pets.

## Data Availability

Not applicable.

## Supplementary Materials

The following supporting information can be downloaded at: https://www.mdpi.com/article/10.3390/toxics11080718/s1, Figures S1–S6: Original image for blots.
