# Supplementary material for: TiO2-Photocatalyst-Induced Degradation of Dog and Cat Allergens under Wet and Dry Conditions Causes a Loss in Their Allergenicity
Source: Toxics. 2023 Aug 21;11(8):718. doi: 10.3390/toxics11080718 (PMC10458468; doi:10.3390/toxics11080718)
Supplement: Supplementary file 1 [file toxics-11-00718-s001.zip › toxics-2539987-supplementary.pdf]

toxics

Supplementary Materials

TiO_2 Photocatalyst Induce Degradation of Dog and Cat Allergens in Wet and Dry Conditions Thereby Losing Allergenicity

Ryosuke Matsuura, Arisa Kawamura, Rizo Ota, Takashi Fukushima, Kazuhiro Fujimoto, Masato Kozaki, Misaki Yamashiro, Junichi Somei, Yasunobu Matsumoto and Yoko Aida

Original Images for Blots

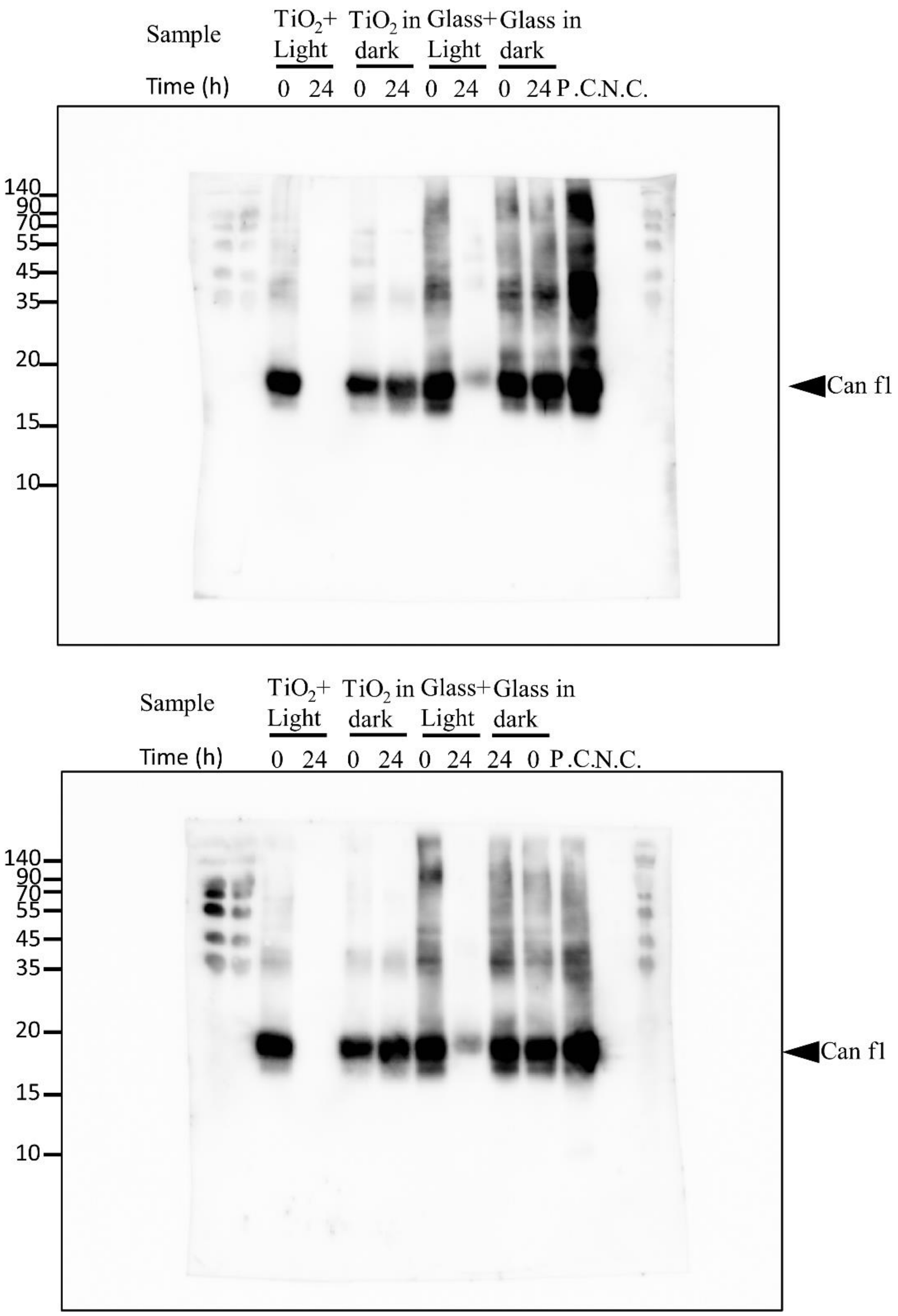

Figure S1. Original Images for Blots of Figure 3 (A) and (B).

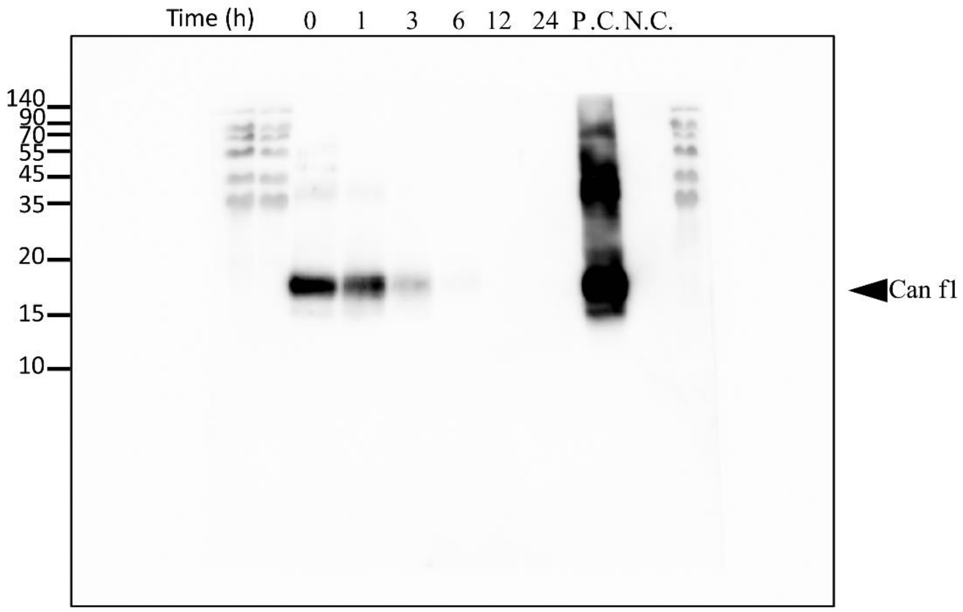

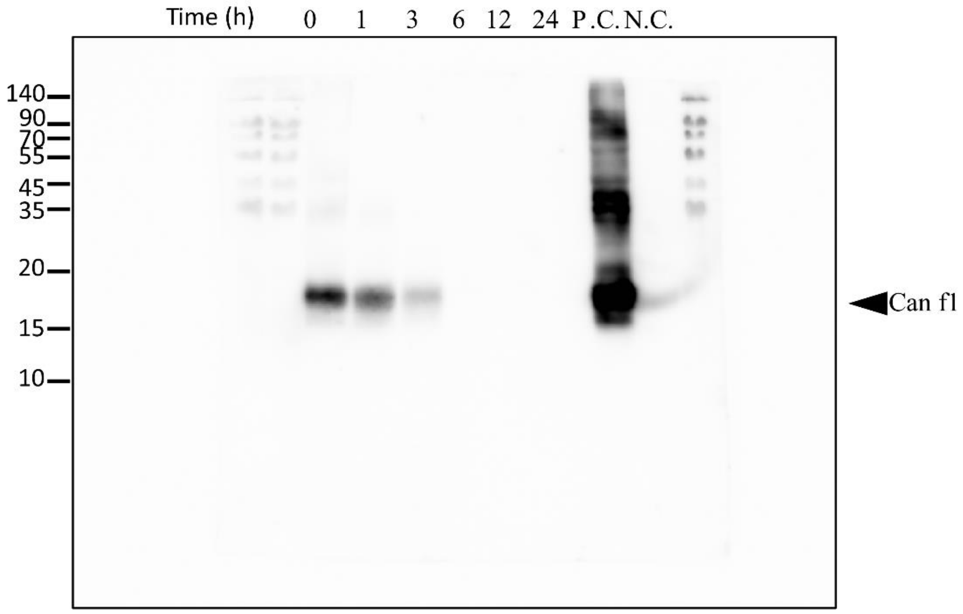

Figure S2. Original Images for Blots of Figure 3 (C) and (D).

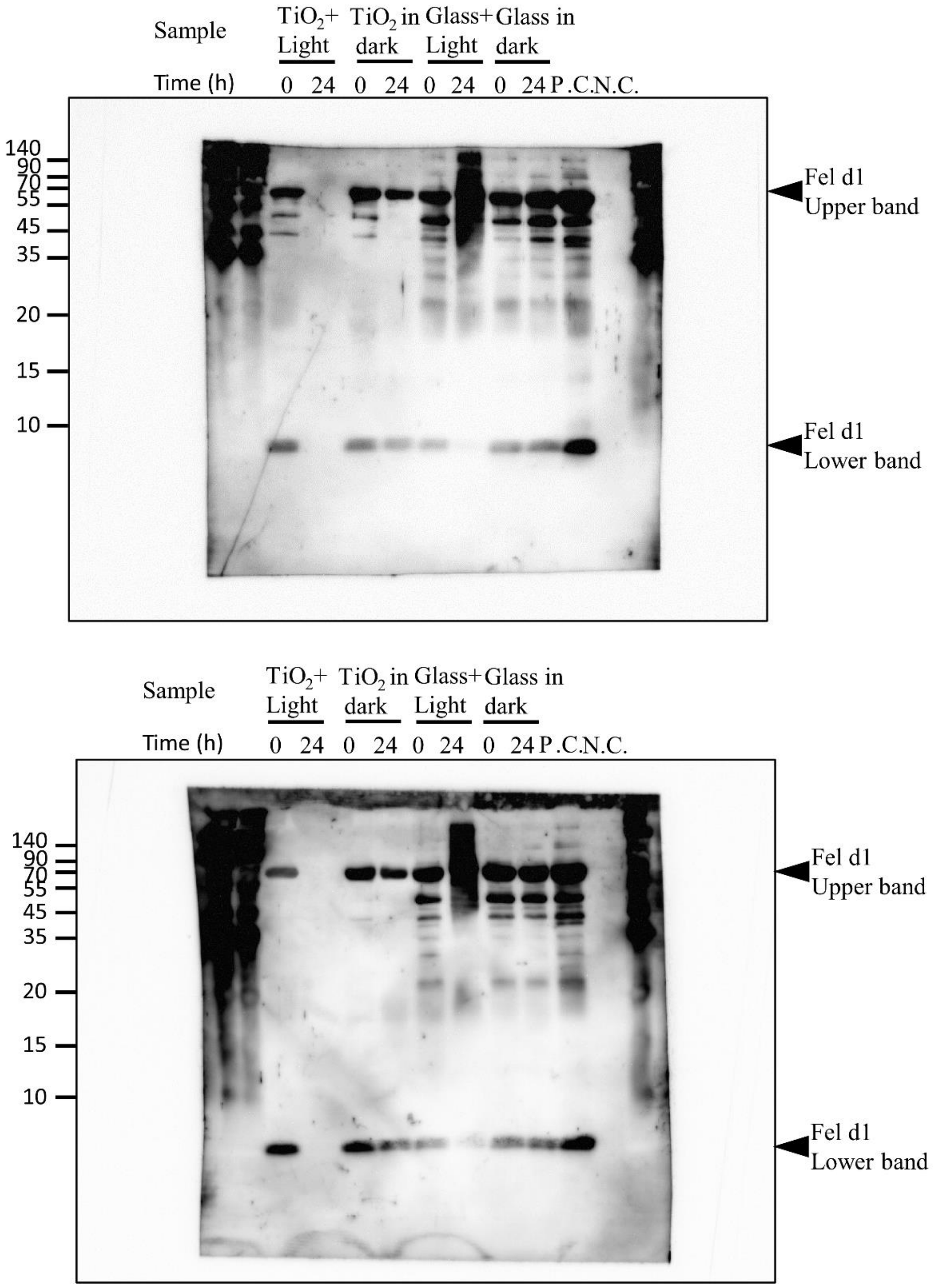

Figure S3. Original Images for Blots of Figure 5 (A) and (B).

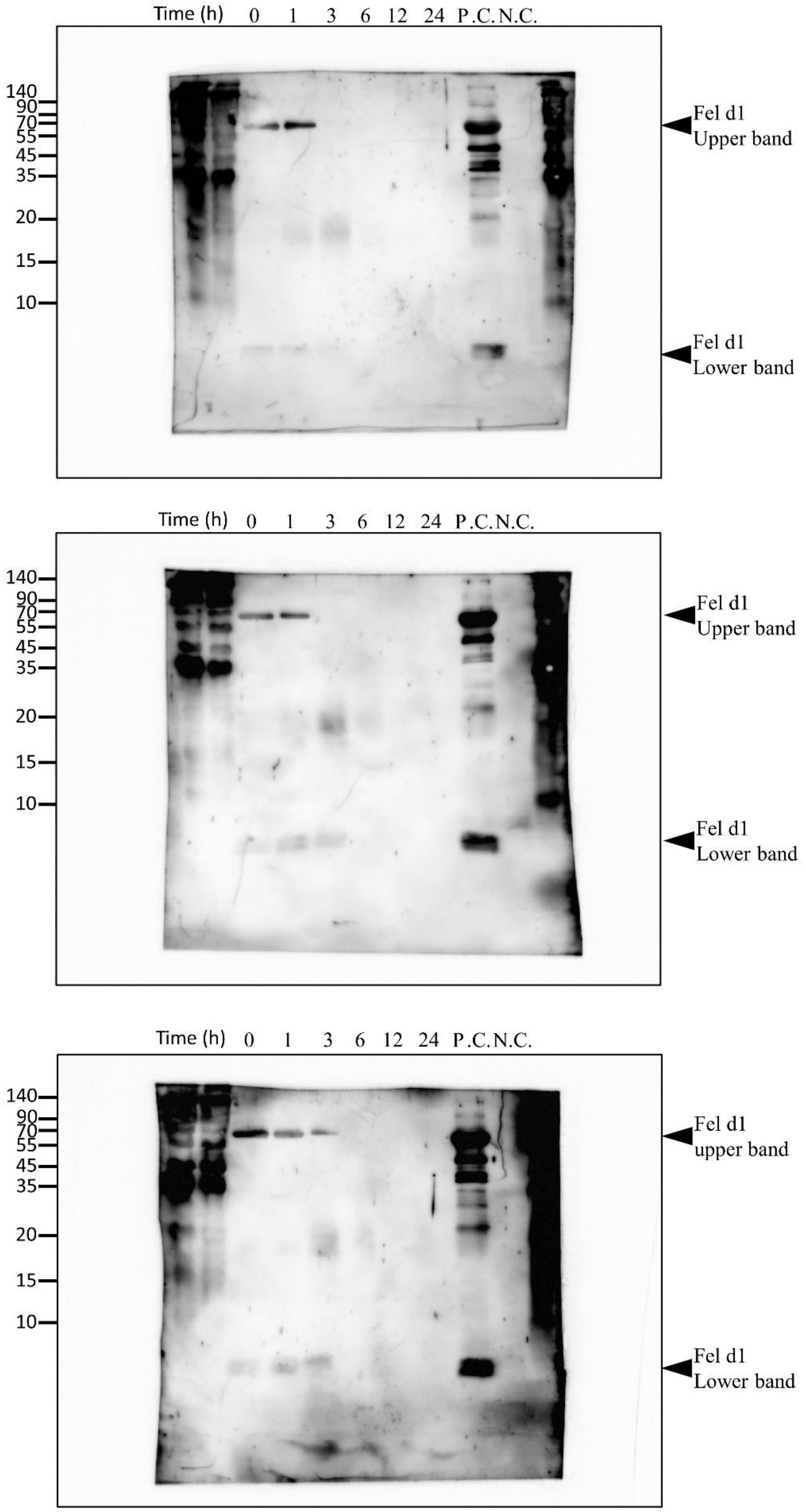

Figure S4. Original Images for Blots of Figure 5 (C) and (D).

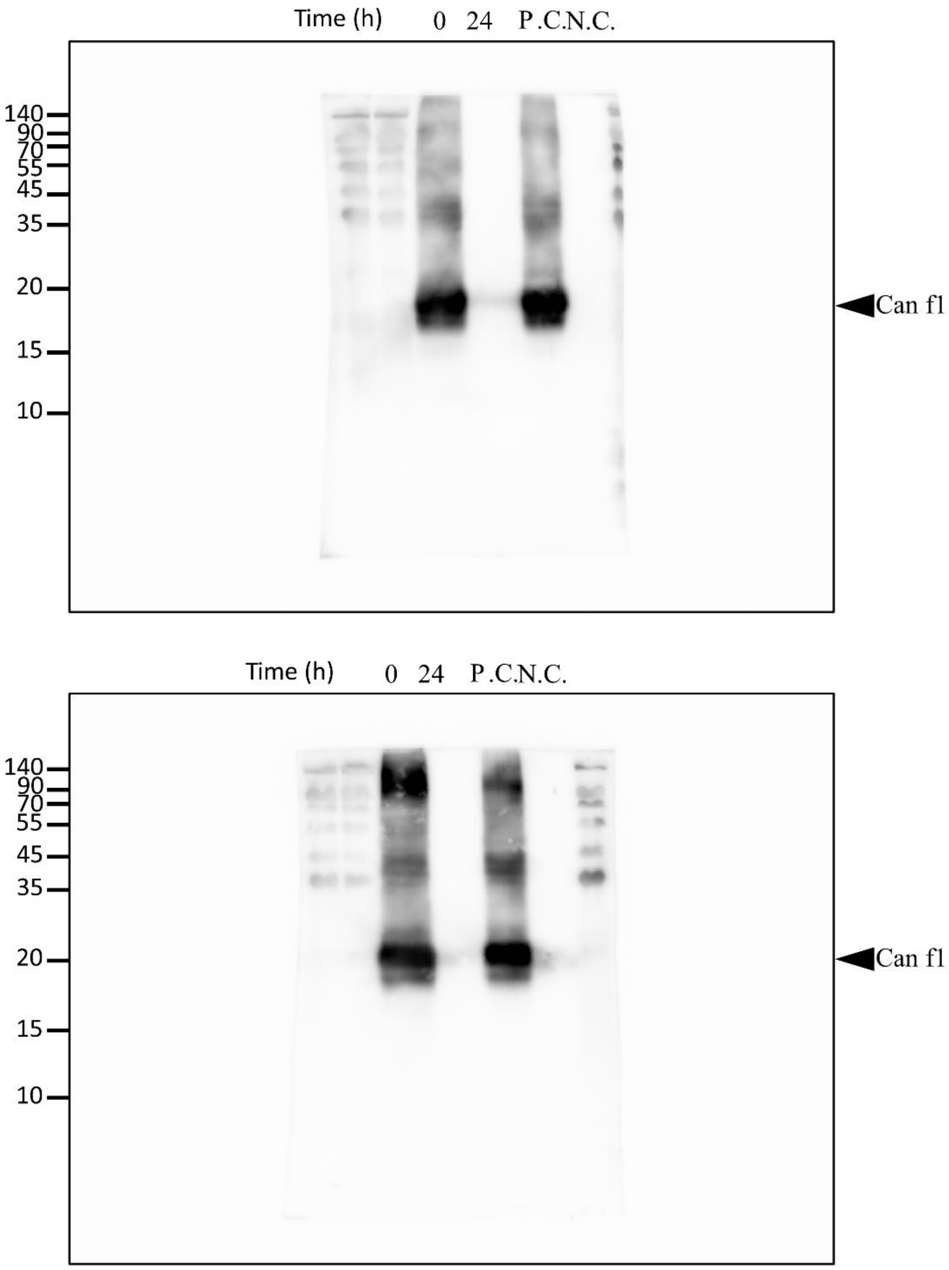

Figure S5. Original Images for Blots of Figure6 (A) and (B).

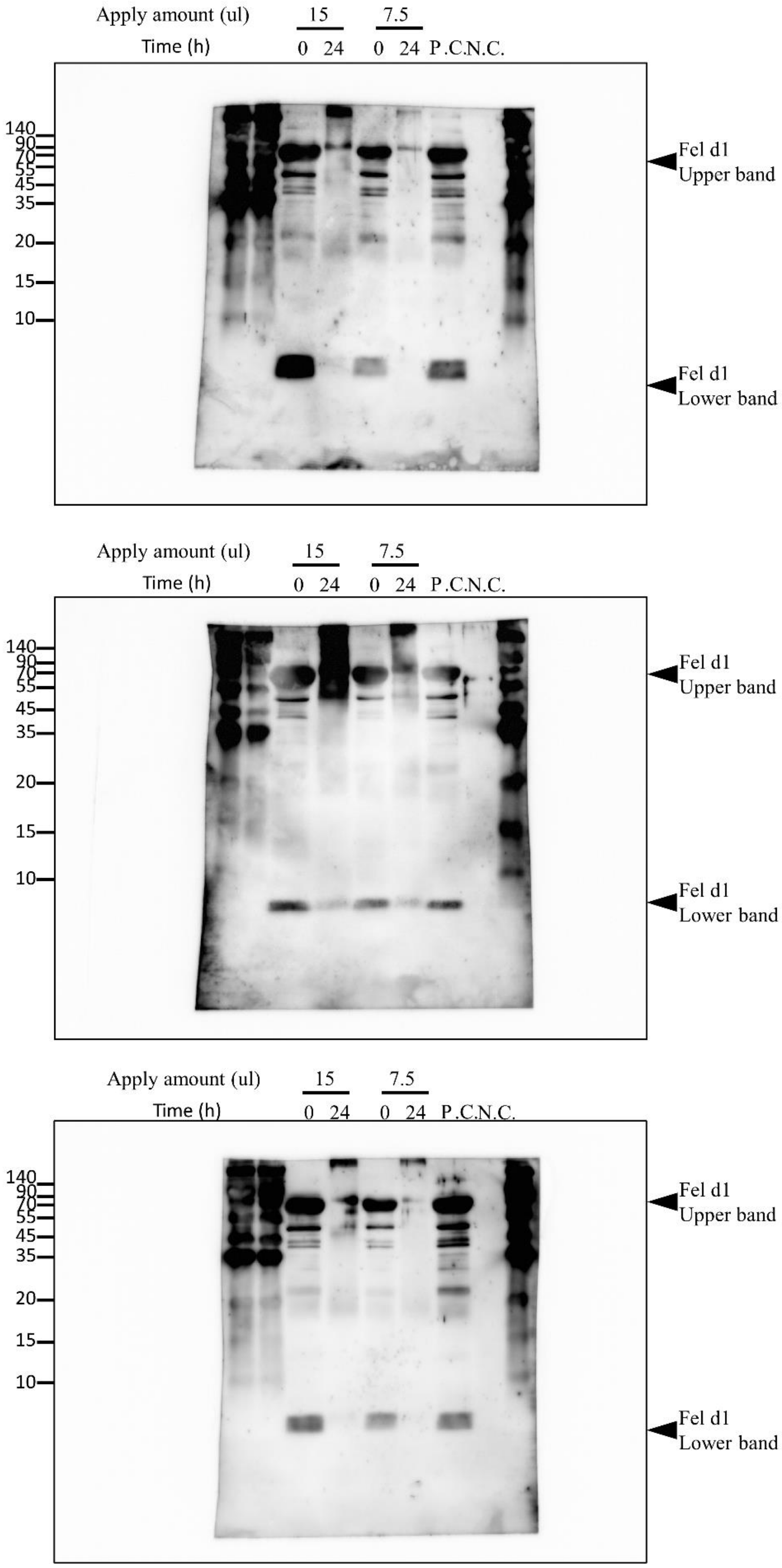

Figure S6. Original Images for Blots of Figure 6 (C) and (D).
